# Comparative performance of a genetically-encoded voltage indicator and a blue voltage sensitive dye for large scale cortical voltage imaging

**DOI:** 10.3389/fncel.2015.00147

**Published:** 2015-04-24

**Authors:** Hiroki Mutoh, Yukiko Mishina, Yasir Gallero-Salas, Thomas Knöpfel

**Affiliations:** ^1^Department of Neurophysiology, Hamamatsu University School of MedicineHamamatsu, Japan; ^2^Laboratory for Neuronal Circuit Dynamics, RIKEN Brain Science InstituteWako City, Japan; ^3^Centre for Global Communication StrategiesThe University of Tokyo, Tokyo Japan; ^4^Division of Brain Sciences, Department of Medicine, Imperial College LondonLondon, UK

**Keywords:** optical imaging, cortex, genetically encoded sensors, voltage-sensitive dye imaging, mouse models

## Abstract

Traditional small molecule voltage sensitive dye indicators have been a powerful tool for monitoring large scale dynamics of neuronal activities but have several limitations including the lack of cell class specific targeting, invasiveness and difficulties in conducting longitudinal studies. Recent advances in the development of genetically-encoded voltage indicators have successfully overcome these limitations. Genetically-encoded voltage indicators (GEVIs) provide sufficient sensitivity to map cortical representations of sensory information and spontaneous network activities across cortical areas and different brain states. In this study, we directly compared the performance of a prototypic GEVI, VSFP2.3, with that of a widely used small molecule voltage sensitive dye (VSD), RH1691, in terms of their ability to resolve mesoscopic scale cortical population responses. We used three synchronized CCD cameras to simultaneously record the dual emission ratiometric fluorescence signal from VSFP2.3 and RH1691 fluorescence. The results show that VSFP2.3 offers more stable and less invasive recording conditions, while the signal-to-noise level and the response dynamics to sensory inputs are comparable to RH1691 recordings.

## Introduction

The use of voltage-sensitive dyes (VSDs) has been developed over the last decades into a powerful tool for monitoring large numbers of neurons simultaneously over large areas of mammalian cortex (Grinvald and Hildesheim, 2004; Frostig, 2009; Canepari, 2011). This approach complements microelectrode electrophysiology that specializes on monitoring smaller number of neurons at single cell level. While mesoscopic VSD imaging covering several mm of cortical area has revealed much information about the spatial organization of cortical representations, it does not resolve the contribution of specific classes of cells such as excitatory and inhibitory neurons. The difficulty in targeting specific cell populations has hindered a refined understanding how defined cell populations are recruited during different stages of information processing.

Genetically-encoded voltage indicators (GEVIs) overcome this limitation as they can be targeted to defined cell populations using molecular biological methods (Knopfel et al., 2006; Knopfel, 2012; Mutoh and Knopfel, 2013). Moreover, the non-invasive staining of neurons with GEVIs greatly facilitates longitudinal studies where imaging is performed in repetitive sessions distributed over days, weeks, and months (Knopfel et al., 2006; Knopfel, 2012; Mutoh and Knopfel, 2013).

Current development of GEVIs focuses on two design principles: (i) Molecular fusion of a voltage sensing domain and a FRET pair of fluorescent proteins (prototypic design of VSFP1/2; Sakai et al., 2001; Dimitrov et al., 2007) or a single fluorescent protein (prototypic design of VSFP3.x; Lundby et al., 2008; Gautam et al., 2009). A large variety of GEVIs are based on this design principle (Tsutsui et al., 2008; Barnett et al., 2012; Jin et al., 2012; Lam et al., 2012; Han et al., 2013; Tsutsui et al., 2013; St-Pierre et al., 2014). (ii) Opsin based GEVIs (prototypic design of Arch; Kralj et al., 2012) that may include readout by a single fluorescent protein (Gong et al., 2014; Zou et al., 2014).

The VSFP family of voltage sensing domain-based GEVIs report membrane voltage changes with response kinetics that are much slower than the microsecond response times of state of the art VSDs such as the “blue dye” RH1691 (Shoham et al., 1999; Akemann et al., 2010). Therefore, early VSFP versions were not optimized for recordings of fast action potentials. This kinetic issue has been resolved with newer voltage sensing domain-based GEVIs such as ASAP1 (St-Pierre et al., 2014). However, cortical population signals captured with VSDs are typically much slower than single action potentials (Petersen et al., 2003a,b; Grinvald and Hildesheim, 2004; Ferezou et al., 2007) and therefore, even relatively slower VSFP variants may be used to replace VSDs with GEVIs for mesoscopic cortical voltage imaging. The FRET based VSFPs such as VSFP2s and VSFP butterflies have been successfully used for this purpose (Akemann et al., 2010, 2012; Mishina et al., 2014). However, the performance of VSFPs has never been directly compared with that of state of the art VSDs under conditions of mesoscopic cortical population voltage imaging.

Here we address this issue and find that even one of the earlier prototypic VSFPs, namely VSFP2.3, performs as well or better than RH1691 in mapping cortical representation of sensory information. Moreover, we demonstrate chronic recordings using VSFP Butterfly1.2.

## Materials and methods

### *In utero* electroporation, surgical procedures, and dye staining

Pregnant ICR mice (CD-1, Japan SLC) were obtained and *in utero* electroporation was performed on E15.5 mouse embryos with pCAG-VSFP2.3 or pCAG-VSFP Butterfly1.2 plasmids. VSFP2.3-expressing mice, 4–24 weeks old, were deeply anesthetized with pentobarbital or urethane as described previously (Akemann et al., 2010, 2012). Body temperature was maintained at 37°C with a heating pad through feedback control (TR-200, Fine Science Tools). The skull was exposed and a metal bolt was attached to the frontal skull with dental cement (Super-Bond C&B, Sun Medical). A transcranial window was made over the somatosensory barrel cortex of one hemisphere by thinning the skull. The window was reinforced and sealed by a thin cover glass (8 mm in diameter) for non-invasive (that is, before VSD staining) imaging.

For simultaneous imaging of the organic voltage sensitive dye (RH1691) and the genetically-encoded voltage sensitive fluorescent protein (VSFP2.3), the thinned bone and dura mater were fully removed over the somatosensory cortex (craniotomy; 6–8 mm in diameter). The cortex was exposed to the VSD RH1691 (0.1 mg/ml, Petersen et al., 2003a) in physiological saline for 1 h. Subsequently, the cortex was washed for 15 min and then covered with 1% agar dissolved in physiological saline. A thin cover glass (8 mm in diameter) was placed on top and stabilized (and sealed) using dental cement. All experiments were conducted according to the Animal Care and Use Committees of RIKEN Brain Science Institute and Japan Neuroscience Society.

### Confocal imaging

At the end of experiments, mice were anesthetized with an overdose of pentobarbital and perfused transcardially with ice-cold artificial cerebrospinal fluid (ACSF; composition in mM: NaCl 118, NaHCO_3_ 25, NaH_2_PO_4_ 1, KCl 3, MgCl_2_ 1, CaCl_2_ 2 and glucose 10), followed by 2% paraformaldehyde in 0.1 M PB (pH 8.0). Brains were removed and post-fixed in the same fixative for 1 h, then transferred to 0.1 M PB. The cortex was sectioned with a Vibratome (VT 1000S, Leica) at 70–100 μm. Coronal slice sections were mounted on an upright microscope using C1si spectral confocal imaging system (Nikon).

### *In vivo* optical imaging

After the recovery from surgical procedures, mice were deeply anesthetized with pentobarbital for initial VSFP2.3 imaging and then with urethane for simultaneous VSFP2.3 and VSD imaging. Dual or triple-channel fluorescence imaging was performed in a system built on a tandem-lens macroscope (THT, Brainvision) with two beam-splitter boxes (DL-FLSP, Brainvision) providing three emission channels. Fluorescence excitation was achieved with two separately shuttered halogen lamps (Moritex), one in epifluorescence configuration (VSFP2.3 excitation) and the other using oblique side illumination (RH1691 excitation). For VSFP2.3 experiments, fluorescence excitation was typically performed at maximum lamp intensity (yielding fluorescence intensities close to saturation levels of the camera), while VSD imaging involved reducing the lamp output to remain below saturation levels of the camera.

**Figure 1:**
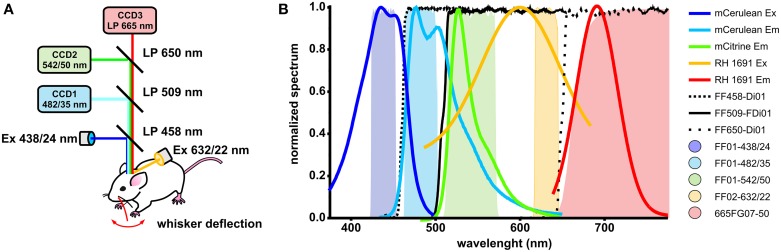
***In vivo* optical voltage recording system, fluorophores spectra and filters and dichroic mirrors transmission spectra. (A)** Schematic drawing of the optical system with three synchronized CCD cameras. Two excitation light sources are used: blue light to excite VSFP2.3 (blue line) is reflected at the first beam splitter, and orange light to excite RH1691 (orange line) was provided by side illumination. **(B)** Normalized excitation and emission spectra of mCerulean, Citrine and RH1691 along with normalized transmission spectra of filters and dichroic mirrors.

Optical signals were acquired with two or three synchronized CCD cameras (Sensicam, PCO). The following optical filters were used: FF01-438/24-25 (Semrock) for mCerulean excitation, FF02-632/22-25 (Semrock) for RH1691 excitation, FF01-482/35-25 (Semrock) for mCerulean emission, FF01-542/50-25 (Semrock) for Citrine emission, 665FG07-50 (Andover Corporation) for RH1691 emission, FF458-Di01-50 × 70 (Semrock) as excitation beam splitter, FF509-FDi01-50 × 70 (imaging-flat dichroic beam splitter, Semrock) as first detection beam splitter, and FF650-Di01-50 × 70 (Semrock) as second detection beam splitter (Figure 1A). Two alternative strategies were employed for triple emission channel imaging: (i) simultaneous fluorescence excitation and recordings of mCerulean, Citrine and RH1691 or (ii) fluorescence excitation and recordings of VSFP2.3 interlaced with RH1691. For chronic VSFP Butterfly1.2 imaging the following filters were used: FF01-483/32-25 (Semrock) for Citrine excitation, FF01-542/27-25 (Semrock) for Citrine emission, BLP-594R-25 (Semrock) for mKate2 emission, FF509-FDi01-50 × 70 (imaging-flat dichroic beam splitter, Semrock) as excitation beam splitter, FF593-Di03 (Semrock) as detection beam splitter. The C1 whisker contralateral to the imaged hemisphere was trimmed to 10 mm in length and other whiskers were cut off and a single air puff (~45 kPa, 100 ms duration) was delivered through picospritzer (Parker) operated by a pulse generator (Master-8, A.M.P.I).

### Data analysis

Optical signals were analyzed using custom programs on Image-Pro Plus 6.2 (Media Cybernetics) and Origin 8 (OriginLab). All data are expressed as mean ± SEM; and *n* represents the number of experiments performed on different mice. For statistical significance of two population means, unpaired and paired two-sample *t*-tests were used, unless otherwise noted.

## Results

### RH1691 staining of VSFP2.3 expressing mice

In this study we used the GEVI VSFP2.3 which has good performance in monitoring neuronal activity in living mice (Akemann et al., 2010). VSFP2.3 uses the fluorescent proteins mCerulean and Citrine that can be combined with the far-red VSD RH1691 as the excitation and emission spectra of RH1691 and VSFP2.3 can be separated by the chosen filter sets (Figures 1A,B). Importantly, the excitation of RH1691 at 630 nm avoids the direct excitation of Citrine and avoids the bleed-through of excitation light into the Citrine emission channel.

**Figure 2:**
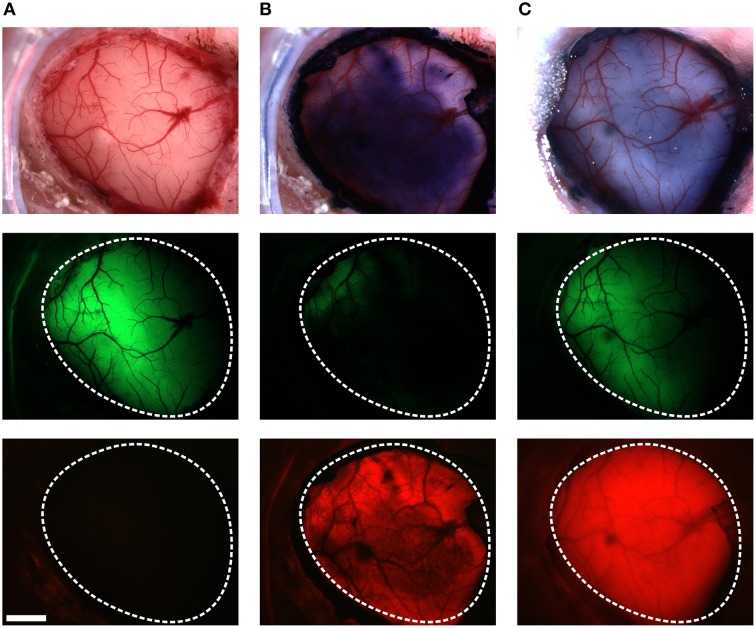
**Staining VSFP2.3-expressing cortex with RH1691**. Brightfield images (top row), Citrine fluorescence (middle row) and red fluorescence (bottom row), before **(A)**, after staining with RH1691 (**B**; 15 min after washing with dye free solution) and the end of the *in vivo* experiment (**C**; 240 min after washing). The white-dashed outlines indicate the area of craniotomy. Note that VSFP2.3 fluorescence was strongly diminished by the RH1691 stain. Scale bar, 1 mm.

**Figure 3:**
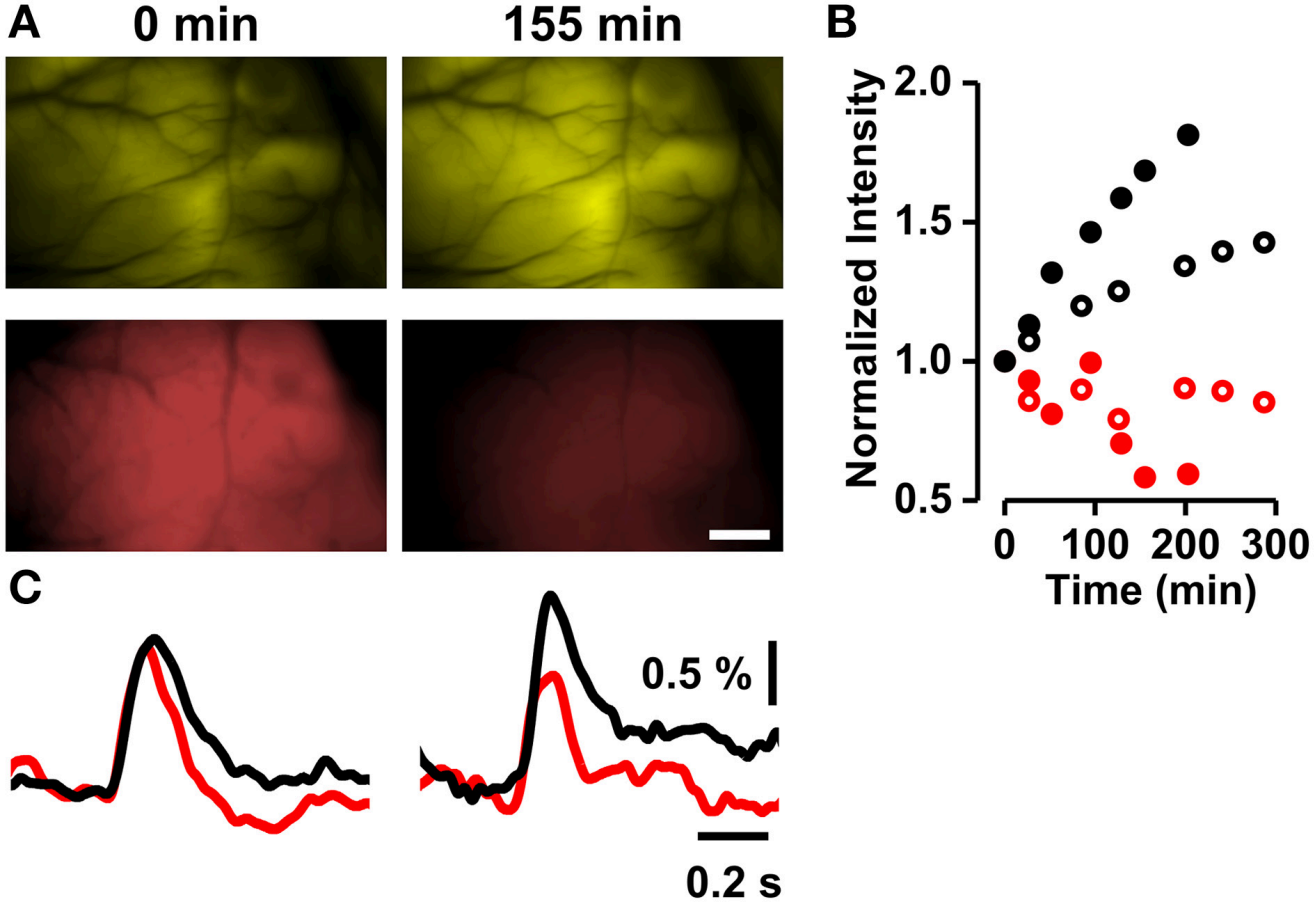
**Destaining of RH1691 and recovery of VSFP2.3 fluorescence. (A)** VSFP2.3 Citrine fluorescence (top row) and RH1691 red fluorescence (bottom row) images taken immediately (left column) and 155 min (right column) after washing off the staining solution. Scale bar, 1 mm. **(B)** Time course of normalized fluorescence intensities (red symbols; RH1691, black symbols; Citrine fluorescence of VSFP2.3; open and closed symbols are from two different animals). **(C)** VSFP2.3 signal (ΔR/R, black) and RH1691 signal (ΔF/F, red) measured at times indicated in **(A)**.

VSFP2.3 expressing mice, prepared with a thinned cranial window several days earlier, were first tested for VSFP2.3 signals evoked by C1 whisker deflection. For these experiments, the responsive areas of the VSFP2.3 signals in the somatosensory cortex were first acquired using the mCerulean and the Citrine emission channels without craniotomy. This protocol allowed us to assess if the VSFP2.3 signals were affected by the subsequent craniotomy and RH1691 staining. In 4 of 10 mice, the VSFP2.3 signals were robustly seen before craniotomy, but too small to be detected after craniotomy and RH1691 staining. We speculate that the main reason for this loss of responsiveness was a low success rate of the duratomy without allowing for recovery from this invasive procedure. Also, VSDs are known to have pharmacological effects and toxicity (Mennerick et al., 2010; Grandy et al., 2012); therefore, even a slight tissue irritation from duratomy can escalate with subsequent RH1691 staining. Animals in which the VSFP2.3 signal was lost were excluded from the comparative analysis. A representative preparation from a successful craniotomy and staining procedure is shown in Figure 2. RH1691 stained the cortical surface to a dark blue coloration (Figure 2B top—middle image). In 6 out of 10 mice, VSFP2.3 signals could be clearly detected after RH1691 staining (Figures 3, **5**), even though the absolute fluorescence intensity of VSFP2.3 had markedly decreased, with the intensity of the Citrine signal decreasing from 1692 ± 235 (12 bit values of the camera) before VSD staining to 639 ± 204 after staining. Notably, light that excites VSFP2.3 and fluorescence emission of VSFP2.3 is absorbed by RH1691 stained tissue (hence the blue coloration). Consistent with this interpretation, the VSFP2.3 fluorescence intensity recovered with wash out of RH1691 over time after the staining (Figure 2, right column).

Thus, over time the intensities of VSFP2.3 and RH1691 fluorescence increased and decreased, respectively (Figures 3A,B). The amplitude of the VSFP2.3 signal increased in parallel with the recovery of its baseline fluorescence, while that of RH1691 degraded over time (Figure 3C).

### Fluorescence of VSFP2.3 and RH1691 in postmortem fixed tissue

**Figure 4:**
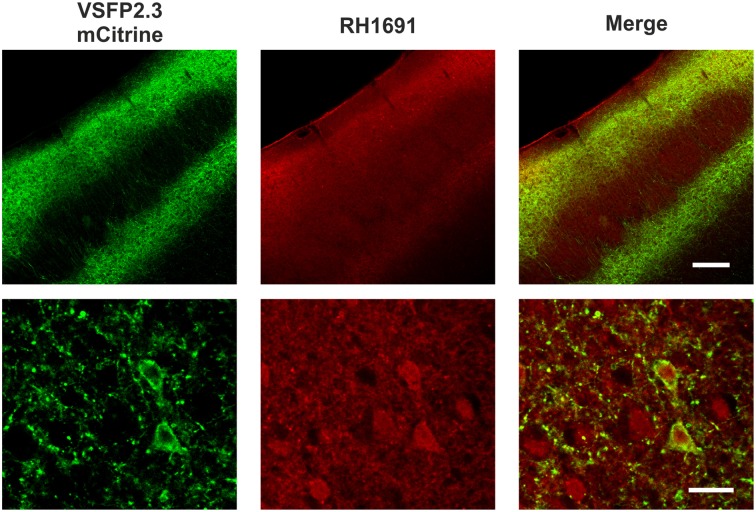
**Distribution of VSFP2.3 and RH1691 fluorescence of fixed coronal sections of the somatosensory cortex**. VSFP2.3 Citrine fluorescence (left column) and RH1691 fluorescence (middle column) and merged image (right column). Upper row low magnification (scale bar, 200 μm), lower row high magnification (layer 2/3; scale bar, 20 μm). Note that VSFP2.3 is localized to membranes of layers 2/3 and 5 pyramidal neurons, whereas RH1691 staining is seen on all cell types in the neocortical area. Cytosolic stain by RH1691 is likely caused by fixation of the tissue.

After the *in vivo* experiments, the distribution of VSFP2.3 and RH1691 was examined in fixed brains (Figure 4). VSFP2.3 labeled pyramidal neurons were found in layers 2/3 and 5, as expected from *in utero* electroporation at E15.5. In contrast, RH1691 stained neurons and glial cells indiscriminately throughout the cortex but it was not found confined to plasma membranes after the fixation. Overall, the fluorescence signals of VSFP2.3 were distributed in populations of pyramidal neurons, while those of RH1691 indicate representation from all cortical cell types including interneurons and glia cells.

### Direct comparison of VSFP2.3 and RH1691 population voltage signals

**Figure 5:**
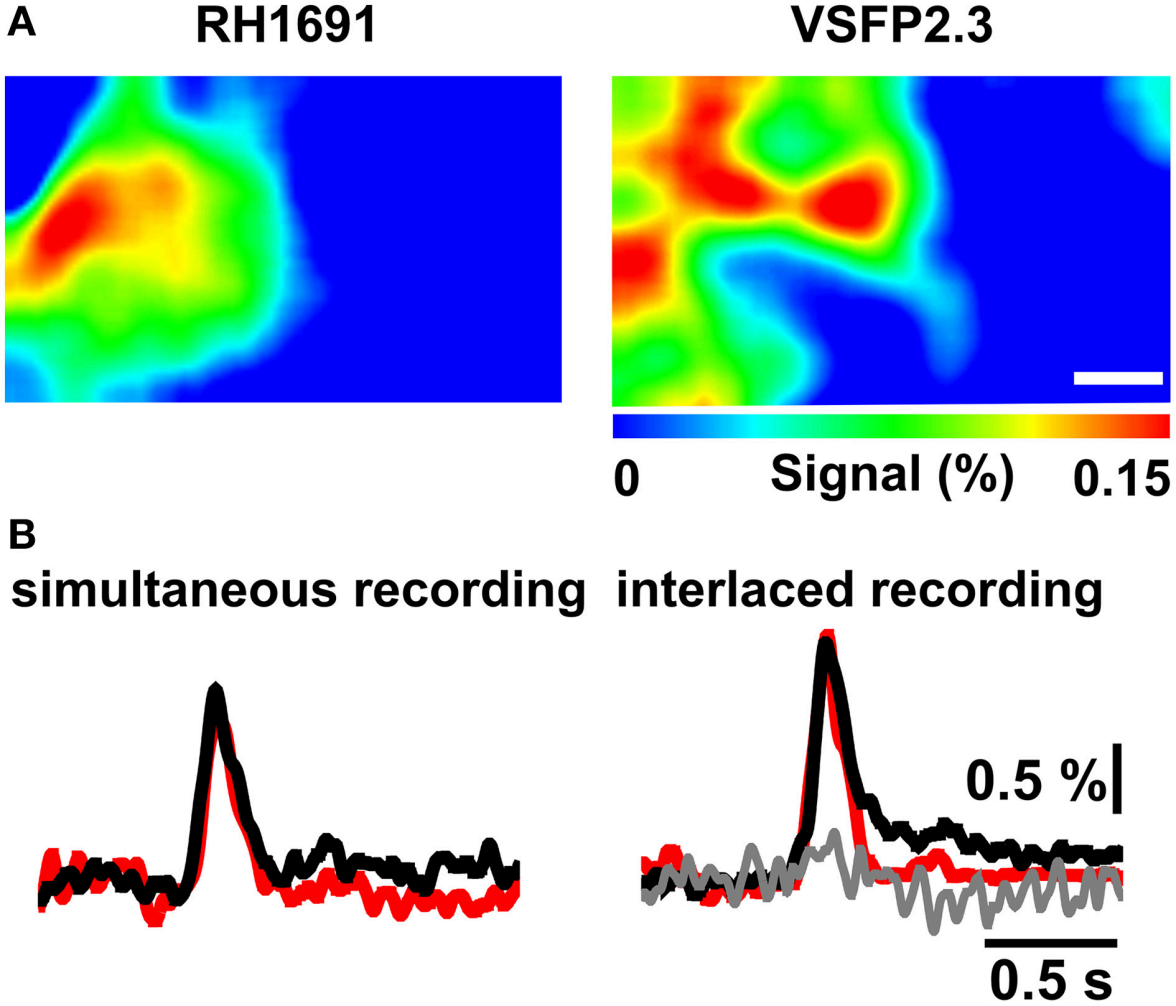
**Combined VSFP2.3 and RH1691 voltage imaging using interlaced and simultaneous recording modes. (A)** Representative peak population membrane voltage signal during a brief C1 whisker deflection imaged with RH1691 (left) and VSFP2.3 (right) using three synchronized CCD cameras (simultaneous recording mode). Scale bar, 1 mm. **(B)** Left VSFP2.3 (black) and RH1691 (red) traces are sampled from **(A)** by a simultaneous recording mode. Right traces are also samples from the same mouse, but acquired using the interlaced VSFP2.3 (black) and RH1691 (red) recording mode. Gray trace is obtained from the RH1691 channel during the VSFP2.3 excitation in the interlaced mode. Note that VSFP2.3 Citrine fluorescence changes do not interfere with RH1691emission. Images and traces represent a 50-trial average.

To compare VSFP2.3 and RH1691 signals, population responses were induced by mechanical deflection of the C1 whisker and optical signals were recorded using the double excitation and triple emission optical configuration (Figure 1A). Initially, VSFP2.3 and RH1691 fluorescence signals were recorded in separate but alternating measurement trails. This interlace strategy eliminates the risk of signal crosstalk while minimizing the possible effect of drifts in recording conditions between individual trails, such as differences in the level of anesthesia. In this mode, VSFP2.3 (black) and RH1691 (red) showed very similar signals (Figures 5A,B right), both in terms of baseline-normalized amplitude and time course. After completion of the recordings in interlaced mode, the simultaneous mode was used where both indicators are imaged at the exact same time. In this simultaneous mode, the signals also appeared to be very similar (Figure 5B left) and there was no evidence of any crosstalk between the VSFP2.3 and the RH1691 signals (Figure 5B right-gray trace).

**Figure 6:**
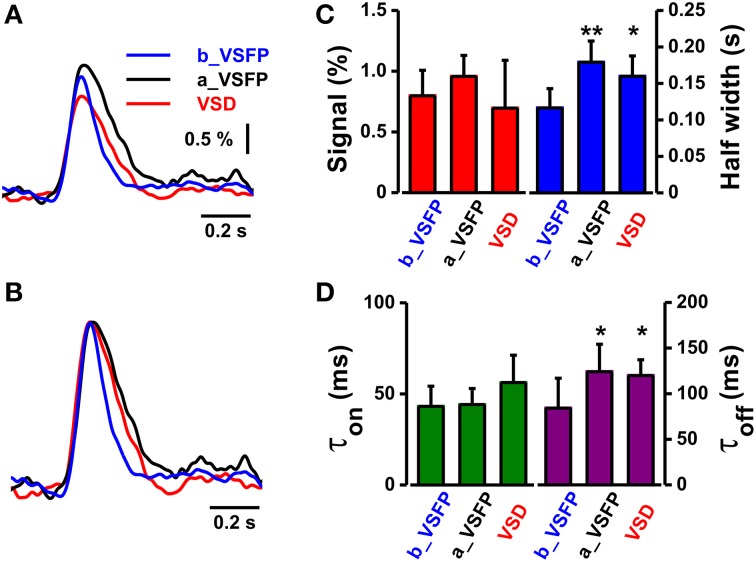
**Comparison of the kinetics of VSFP2.3 and RH1691 signals induced by C1 whisker deflection. (A)** Average VSFP2.3 and RH1691 signals from five mice: VSFP2.3 signals before RH1691 staining (blue, b_VSFP), VSFP2.3 signals after RH1691 staining (black, a_VSFP), and RH1691 signals (red, VSD). **(B)** Data from **(A)** normalized to peak amplitude. **(C)** Comparison of the mean signal amplitudes and widths (at half-maximum) taken from the voltage signals in **(A)** (mean ± sem). *T*-tests were performed for a_VSFP and VSD, both against b_VSFP; ^*^P < 0.05, ^**^P < 0.005. **(D)** Comparison of the mean on-time constant (τ_on_) and decay-time constant (τ_off_) taken from the normalized voltage signals in **(B)**. *T*-tests were performed for a_VSFP and VSD, both against b_VSFP; ^*^P < 0.05, ^**^P < 0.005.

Comparison of the performance of both indicators under post craniotomy condition revealed a similar time course of the VSFP2.3 and RH1691 C1 whisker deflection-induced optical signals (VSFP2.3 signal after VSD staining; amplitude 0.96 ± 0.17%, half-width 0.18 ± 0.03 s, τ_on_ 43 ± 11 ms, τ_off_ 124 ± 30 ms, VSD; amplitude 0.70 ± 0.4%, half-width 0.16 ± 0.03 s, τ_on_ 56 ± 16 ms, τ_off_ 120 ± 17 ms, *n* = 6). Averaged amplitudes of VSD signals tend to decrease due to bleaching of signals but this does not occur in VSFP2.3 signals. However, the VSFP2.3 signals obtained prior to the craniotomy were clearly faster than the VSFP2.3 and RH1691 signals that followed craniotomy and dye loading (Figures 6A–D, VSFP2.3 signal before VSD staining; amplitude 0.79 ± 0.21%, half-width 0.12 ± 0.03 s, τ_on_ 43 ± 11 ms, τ_off_ 80 ± 33 ms, *n* = 6).

### Chronic *in vivo* experiments in Butterfly1.2 electroporated mice

Genetically-encoded fluorescent indicators greatly facilitate longitudinal studies where imaging needs to be performed in repetitive sessions distributed over days, weeks, or even months. Although expected from the above transcranial imaging approach and embryonic gene transfer, chronic imaging of GEVIs has not yet been well documented.

**Figure 7:**
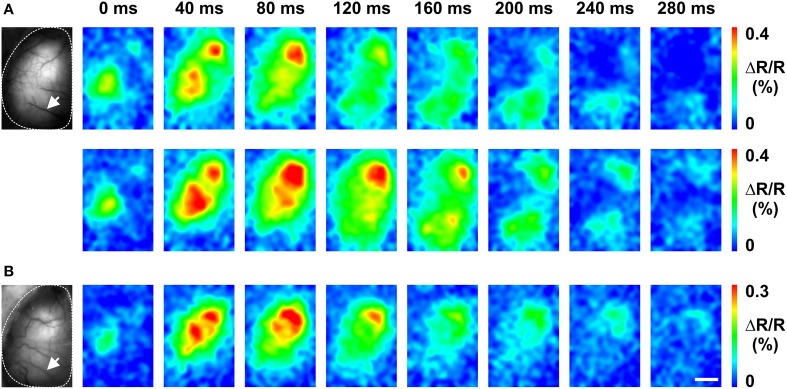
**Chronic *in vivo* imaging in the somatosensory cortex using VSFP Butterfly1.2. (A)** Baseline Citrine fluorescence image over left cortical hemisphere of P127 mouse (left) and time series of C1 whisker deflection-induced membrane voltage signals (right). Upper and lower row show two different measurements (each representing the average over 50 trails) recorded on the same day. **(B)** Imaging session performed at P176, otherwise as in **(A)**. White arrows in **(A,B)** indicate the same blood vessels. The transcranial window was prepared at P120. Scale bar, 2 mm.

To this end, Butterfly1.2 was used. Figure 7 illustrates an experiment with an *in utero* electroporated mouse that was prepared for imaging at postnatal day 120 (P120) with a large transcranial window. In the first imaging session performed after several days of recovery and under anesthesia, C1 whisker deflection showed a butterfly signal that was initially confined to the somatosensory cortex with spread into the whisker motor cortex. Over the following tens of milliseconds, these signals spread to large portions of the hemisphere (Figure 7A upper). Upon repetition (Figure 7A bottom), the sensory stimuli-induced population voltage signals displayed a similar pattern of propagation. A second imaging session was performed more than a month later at P176. Although the bone around the transcranial window showed some regrowth and some new blood vessels appeared, C1 whisker deflection induced similar population voltage signals and the same propagation pattern (Figure 7B).

## Discussion

The present study was motivated by the recent promising advances in GEVI developments and the question how fast a voltage indicator needs to be so that it can substitute VSDs in mesoscopic cortical voltage imaging. In this context it should be noted that the most recently developed GEVIs allow us to monitor action potentials and oscillatory membrane voltage responses at frequencies of up to 200 Hz, albeit they are assessed mostly only in cultured cells or acute brain slices (Gong et al., 2014; Hochbaum et al., 2014; St-Pierre et al., 2014) and do not provide a ratiometric signals that is crucial for correcting hemodynamic components of the optical signals. Moreover, imaging technologies that would allow to image from large number of cells at millisecond temporal resolution (e.g., frame rates of 1 kHz) in living rodents are not yet readily available.

Although voltage imaging at the level of single cells has contributed to many interesting insights, the more unique and more powerful application of voltage imaging has been at the mesoscopic scale, covering large cortical areas (Grinvald and Hildesheim, 2004). The classical VSD approach to mesoscopic cortical voltage imaging using dyes such as RH1691 exhibits technical limitations related to the need for a full craniotomy for staining the brain with the VSD, which can have by its own a negative impact on brain function. In addition, VSDs are known to have pharmacological effect including actions on inhibition (Mennerick et al., 2010; Grandy et al., 2012). In our hands, swelling of the brain surface was commonly observed after craniotomy and removal of the dura matter. This, along with a possible pharmacological effect may explain the prolonged voltage signal recorded using both VSFP2.3 and RH1691 after craniotomy and VSD staining (Figure 6). In contrast, transcranial brain imaging using GEVIs (the genes of which are introduced during the fetal period or as transgenes) does not require a craniotomy, leaving the brain in a physiological condition. Notably, GEVIs allow to record brain activities through the fully intact skull by removing only the skin (unpublished data). At present voltage sensing domain based (VSFP-type) GEVIs are the only voltage indicators that have shown sufficient performance in functional brain imaging *in vivo* (Akemann et al., 2010, 2012, 2013). The potential of these GEVIs have been demonstrated in several recent studies of cortical representation of sensory information and state-dependent spontaneous brain activities in anesthetized and awake mice (Scott et al., 2014; Carandini et al., 2015).

The stable preparation of GEVIs in this study also allowed for experiments to be performed for longer than a month (Figure 7), whereas the VSD was able to report for only around 2 h because of washout and photo-bleaching. The VSD signals attenuated and the VSD fluorescence intensity reduced by half in course of a 2 h experimental session. However, in contrast, the VSFP did not show photo-bleaching at the excitation intensity level used. The signal and intensity of GEVIs remained the same at all times unless there were changes caused by the shift of brain state due to the anesthesia level (Scott et al., 2014).

Our results show that GEVIs provides more stable and non-invasive recording conditions, although the signal-to-noise level and function of GEVIs is similar to that of VSD. With the recent generation of transgenic mouse lines that express GEVIs at appropriate levels (Madisen et al., 2015), GEVI-based imaging approaches will accelerate the interpretation of brain activities in living animals.

### Conflict of interest statement

The authors declare that the research was conducted in the absence of any commercial or financial relationships that could be construed as a potential conflict of interest.
